# Supplementary material for: Complement C5 is not critical for the formation of sub-RPE deposits in Efemp1 mutant mice
Source: Sci Rep. 2021 May 17;11:10416. doi: 10.1038/s41598-021-89978-8 (PMC8128922; doi:10.1038/s41598-021-89978-8)
Supplement: Supplementary file 1 — Supplementary Information. [file 41598_2021_89978_MOESM1_ESM.pdf]

Supplementary Information

Title: **Complement C5 is not critical for the formation of sub-RPE deposits in *Efemp1* mutant mice.**

Authors: *Donita L. Garland[1], Eric A. Pierce[1], and Rosario Fernandez-Godino[1]*.*

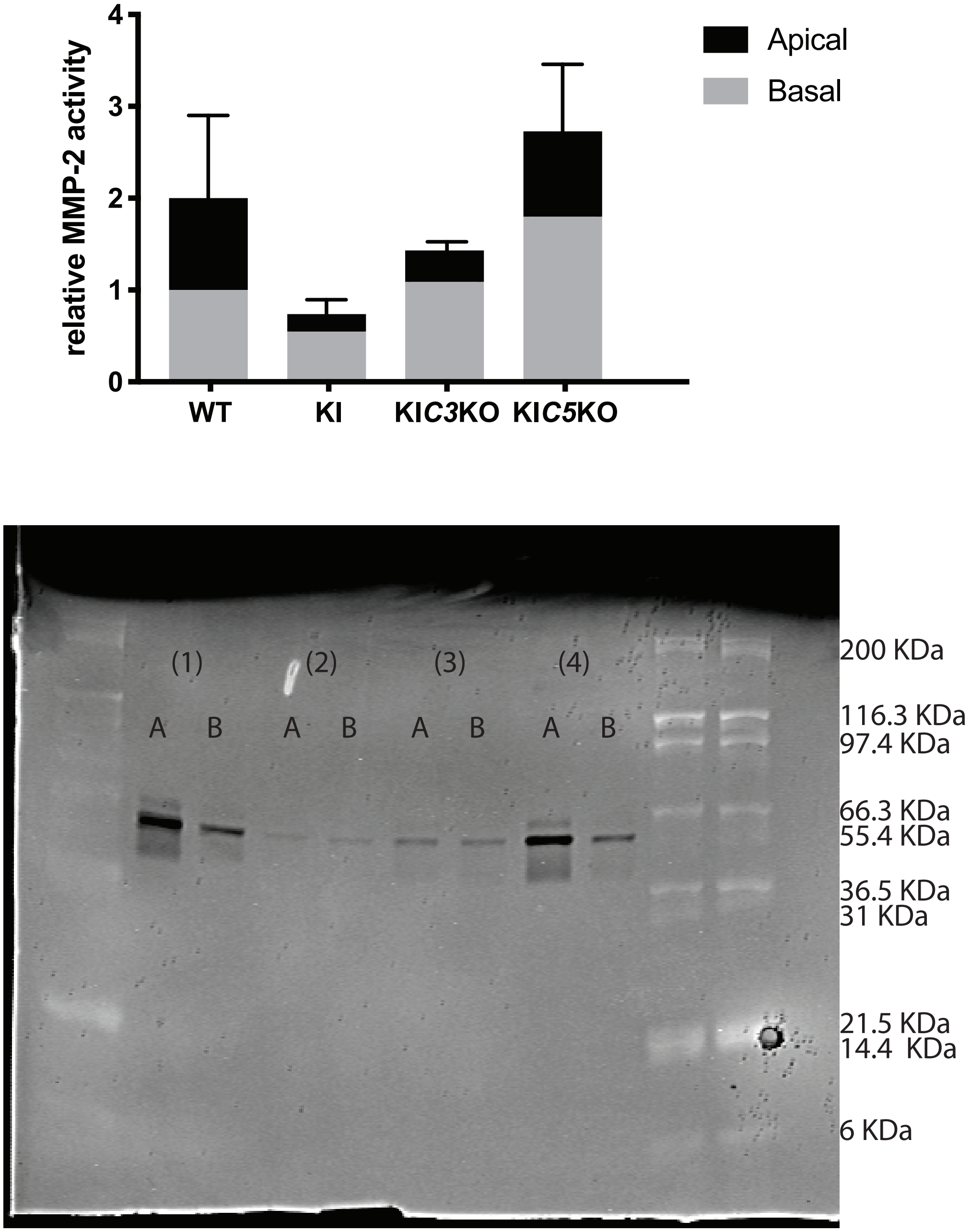

Supplementary figure 1. Quantificatin of the MMP-2 activity measured by zymography in apical (A) and basal (B) conditioned media of (1) *Efemp1*$^{WT/WT}$:*C5*$^{+/+}$, (2) *Efemp1*$^{R345W/R345W}$:*C5*$^{+/+}$, (3) *Efemp1*$^{R345W/R345W}$:*C3*$^{-/-}$ and (4) *Efemp1*$^{R345W/R345W}$:*C5*$^{-/-}$ cultures. The first and two last lanes were loaded with Mark12 unstained standard (Thermo Fisher). Values were normalized to apical and basal MMP-2 activity in wild type samples.
